# Effect of Exposure to Positive Images of Dentistry on Dental Anxiety among 7 to 12 Years Old Children

**DOI:** 10.5005/jp-journals-10005-1260

**Published:** 2015-02-09

**Authors:** Rini Rajendra Gangwal, Sourabh Rameshchandra Badjatia, Bhavna Harish Dave

**Affiliations:** Senior Lecturer, Department of Pedodontics and Preventive Dentistry, Modern Dental College, Indore, Madhya Pradesh, India; Senior Lecturer, Department of Public Health Dentistry, Modern Dental College and Research Centre, Indore, Madhya Pradesh, India; Professor and Head, Department of Pedodontics and Preventive Dentistry, KM Shah Dental College and Hospital, Vadodara, Gujarat, India

**Keywords:** Dental anxiety, Positive images, Venham picture test.

## Abstract

**Aim:** To evaluate the effect of exposure to positive images of dentistry on dental anxiety among 7 to 12 years old children.

**Materials and methods:** Controlled trial. Assessment of anxiety and analysis of data were conducted blind to experimental condition. Assessment of anxiety was carried out in the waiting room prior intervention, postintervention into the operatory during the treatment and again after the completion of treatment. Anticipatory anxiety was recorded by Venham's picture test (VPT).

**Participants:** Sixty children of 7 to 12 years age group.

**Intervention:** Participants were randomly assigned to one of two conditions. In both conditions the participant was asked to look at photographs for 2 minutes in the waiting area prior to their appointment. The intervention consisted of viewing positive images of dentistry and dental treatment (study group), the (control group) consisted of neutral images. The assessment of anticipatory dental anxiety was made blind to experimental condition and statistical analysis was conducted blind to group membership. Anticipatory anxiety assessed by the VP T.

**Results:** A total of 60 subjects participated in the study and were equally and randomly allotted to study group (positive image) and control group (neutral image). The mean anxiety score found at waiting area before intervention, after intervention (OPD) and postoperative was statistically significant in study group. Post hoc comparison of anxiety score in study group showed high statistical significance.

**Conclusion:** Positive dental images have an effect on reducing anxiety as compared to neutral images when measured by the VPT.

**How to cite this article:** Gangwal RR, Badjatia SR, Dave BH. Effect of Exposure to Positive Images of Dentistry on Dental

Anxiety among 7 to 12 Years Old Children. Int J Clin Pediatr Dent 2014;7(3):176 -179.

## INTRODUCTION

Behavioral sciences have become an increasingly important component of dental education and research. Role of behavioral sciences is increasing in application of psychological methods to the study of behavior and attitudes relevant to health, illness and healthcare in particular, fear of dentists and dentistry as well as of dental pain.^1^ Dentistry brings fear and it afficts a major portion of population, which is a menace commonly in children. Fear of clinicians and dentistry is a common and potentially distressing problem.^2^ Dental fear is associated with the reduced oral health status, poorer oral health-related quality of life, and compromised psychosocial health, such as lower self-esteem and lower morale.^3^ One approach known as modeling given by Kuhn and Allen, may be to develop positive associations with dentistry through the promotion of positive images of children experiencing dental treatment. Such an approach draws on the principles of social learning, proposing that exposure to positive images will trigger the learning and association between the positive image and dentistry and is Akin to modeling.^4^

### Aim

To evaluate the effect of exposure to positive images of dentistry on dental anxiety among 7 to 12 years old children

### Objectives

To find out anticipatory anxiety level in children before intervention and determine the impact of positive images and neutral images on dental anxiety of children.

To compare anticipatory anxiety level in children before intervention, after intervention and post dental treatment.

## MATERIALS AND METHODS

The present controlled trial was conducted to evaluate the effect of exposure to positive images of dentistry on dental anxiety among 7 to 12 years old children attending the OPD of pedodontics and preventive dentistry. Prior permission and informed consent were obtained from the parents of children. The study protocol was reviewed and approved by the research cell of KM Shah Dental College and Hospital, i.e. Human Research Review Board and ethical approval was obtained by the ethics committee, Sumandeep Vidyapeeth, Piparia, Vadodara.

### Inclusion Criteria

- Children aged 7 to 12 years attending outpatient department of pedodontics and preventive dentistry.
- Children who needed single tooth extraction.

### Exclusion Criteria

- Children who or whose parents refused to give informed consent
- Children with learning difficulties who were judged unable to understand the instructions
- Children with visual, hearing or speech impairment
- Children that became upset at viewing the images.

### Sample Size

A total of 60 subjects were selected.

The selected samples were divided equally into two groups by lottery method randomly.

*Group 1*: Subjects were shown positive images of dentistry (study group)

*Group 2*: Subjects were shown neutral images (control g r o up).

## INTERVENTION

- *Positive images*: Four photographs of A4 size were chosen by the researcher, associating dentistry with familiar and happy images (these were: three children aged between 7 and 15 years smiling whilst sitting in the dental chair; a Teddy bear sitting in dental chair; a child aged 10 smiling; child aged 5 holding a large toothbrush), with the aim of showing dentistry in a positive light.
- *Neutral images*: Four photographs of A4 size were selected from the most recent issue of a commercially produced magazine related to houses and decorations.
- The subject on arrival was first allocated randomly either to study group or control group and masking was done. The anxiety assessment of the child was conducted by the principal researcher who was blind to the patient's group allocation.
- The patient was allotted 2 minutes to look at the photographs and allowed to choose one how they feel and anxiety was assessed by Venham Picture Test (CPT) children which presents eight pairs of pictures, each depicting cartoon characters in anxious or non-anxious states.
- Assessment of anxiety was done in the waiting area prior to intervention by VPT. Positive images in the form of photographs of dental situations were shown to group 1 children in waiting area and neutral images were shown to group 2 children and their pretreat-ment anticipatory anxiety were recorded by VPT.
- Assessment of anxiety was again carried out in the operatory during the treatment (extraction) and again after the completion of treatment (extraction) by show-i ng VP T.
- All the subjects participated in the study were given treatment for which single tooth extraction which was performed under local anesthesia. Extraction was performed by chief investigator.

## MASKING

The researcher assessing the level of anticipatory anxiety of the children was masked to the children's membership of the group except the researcher who allotted the subjects into groups. Principal researcher was masked who performed the extraction. Children, their parents and the data analyst were also masked. The assessment of anticipatory dental anxiety was made blind to experimental condition and statistical analysis was conducted blind to group membership.

## STATISTICAL ANALYSIS

The survey data so obtained from the selected sample was compiled, systematized, tabulated and master sheet was prepared. Analysis was carried out using SPSS package version 14 (trial version). Friedman ANOVA test, Chi-square test and Wilcoxon signed ranks test was applied. Level of significance was kept at 5% for statistical analysis.

## RESULTS

A total of 60 subjects who participated in the study were equally and randomly allotted to group 1: study group (positive image) and group 2: control group (neutral i m age).

Table 1 illustrates the mean anxiety score found at waiting area, after intervention (OPD) and post-operative which was 6.10 ± 0.80, 4.50 ± 0.77 and 3.70 ± 0.70 respectively. The difference between these value was highly statistically significant p-value = 0.00 (Friedman test, Chi-square = 53.2).

Table 2 shows the difference in anxiety score before intervention, after intervention and postoperative in case group which was highly statistically significant p-value = 0.000 (Wilcoxon Signed Ranks Test).

Table 3 projects distribution of mean anxiety score found at waiting area, After intervention (OPD) and postoperative which was 5.97 ± 0.76, 5.87 ± 0.73 and 6.00 ± 0.69 respectively. The difference between these value was not statistically significant p-value = 0.307 (Friedman test, Chi-square = 0.307).

Table 4 projects the difference in anxiety score before intervention, after intervention and postoperative in control group which was not statistically significant (p-value = 0.083, 0.302 and 0.157 respectively; Wilcoxon Signed Ranks Test).

Table 5 shows comparison of anxiety score seen at Waiting Area which was not statistical significant (p = 0.503; Mann-Whitney test) but was highly statistically significant after intervention (OPD) and postoperatively (p = 0.000 Mann-Whitney test).

**Table 1:** **Table 1:** Distribution of anxiety scores using VPT before intervention, after intervention and after dental exam in study group

*Place*		*Subjects (N)*		*Mean ± SD*		*Chi-square*		*p-value (Friedman ANOVA test)*	
Waiting area		30		6.10 ± 0.80					
After intervention (OPD)		30		4.50 ± 0.77					
						53.2		0.00	
Postoperative		30		3.70 ± 0.70					

**Table 2:** **Table 2:** Distribution of anxiety scores using VPT before intervention, after intervention and after dental exam in study group (Post hoc case)

*Place*		*Subjects (N)*		*Wilcoxon signed rank test*			
Waiting area to after intervention (OPD)		30		Z = –4.817		p = 0.000	
Waiting area to postoperative		30		Z = –4.852		p = 0.000	
After intervention (OPD) to postoperative		30		Z = –4.021		p = 0.000	

**Table 3:** **Table 3:** Distribution of anxiety scores using VPT before intervention, after intervention and after dental exam in control group

*Place*		*Subjects (N)*		*Mean ± SD*		*Chi-square*		*p-value (Friedman ANOVA test)*	
Waiting area		30		5.97 ± 0.76					
After intervention (OPD)		30		5.87 ± 0.73					
						2.364		0.307	
Postoperative		30		6.00 ± 0.69					

## DISCUSSION

Fear of dentistry is common in children. The anxiety that accompanies dental treatment is considered a universal problem and has aggravated the interest of a large number of researchers Aartman et al,^5^ Ramos-Jorge et al,^6^ Olumide et al,^7^ Gustafsson et al,^8^ Sjogren et al.^9^ A number of instruments are available to measure dental anxiety in children.^10^ The Venham picture scale is widely used and easily administered.^11^

In the present study, the effect of positive dental images differed from that of neutral images in reducing anxiety, as measured by VPT, which rejected the null hypothesis. The potential biases pointed out by Fox and Newton, (2006)^4^ were controlled. The study and control groups were paired with regard to their anxiety for dental treatment.

With the exception of the researcher who allocated the children to the different groups, all other members of the research team were masked to the allocation of the participants to the neutral and positive groups. Special care was taken to ensure that the behavior of the researcher who allocated the participants was similar when dealing with the children in both groups. Thus, this researcher was directly observed by another member of the team throughout the entire collection of the data. The observing researcher was unable to distinguish whether the researcher was with the positive group or the neutral group.

**Table 4:** **Table 4:** Distribution of anxiety scores using VPT before intervention, after intervention and after dental exam in control group (Post hoc control)

*Place*		*Subjects (N)*		*Wilcoxon signed rank test*			
Waiting area to after intervention (OPD)		30		Z = –1.732		p = 0.083	
Waiting area to Postoperative		30		Z = –0.302		p = 0.302	
After intervention (OPD) to postoperative		30		Z = –1.414		p = 0.157	

**Table 5:** **Table 5:** Distribution of anxiety scores using VPT before intervention, after intervention and after dental exam in study group and control group

*Place*		*Subjects (N)*		*Mann-Whitney test*			
Waiting area		60		Z = –0.669		p = 0.503	
After intervention (OPD)		60		Z = –5.454		p = 0.000	
Postoperative		60		Z = –6.602		p = 0.000	

In the previous study Fox and Newton (2006)^4^ the assessment of anxiety was performed only one time (after the intervention). Therefore, this study did not determine whether the difference found in the degree of anxiety was present prior to the intervention. To control this problem in the present study, anxiety was also assessed before the intervention.

In the present study, the children who were exposed to neutral images before intervention, after intervention and posttreatment showed no modification in the anxiety level. Children showed virtually the same anxiety level during the whole dental visit. Children who were exposed to positive images before intervention, after intervention and post dental treatment showed signi-ficant difference. High anxiety level reduction was seen in these children.

According to Olumide et al (2009),^7^ it was observed that anxiety scores immediately following a dental examination were higher when the child was submitted to treatments other than a simple dental exam. This is similar to the findings of the present study, where anxiety scores increased immediately after the dental exam in both the groups. Anxiety scores were decreased among subjects in study group who were shown positive images of dentistry which may have been represented as part of modeling and building children positive toward dentistry.

Hence, it was seen that the effect of positive dental images showed difference from that of neutral images in reducing anxiety. Similar to findings of Fox and Newton (2006)^4^ who showed viewing positive images of dentistry and dentists results in short-term reductions in anticipatory anxiety in children. Also, these findings were in contrast to, Ramos-Jorge ML et al (2011)^12^ who showed no difference in the anxiety levels of children exposed to positive image and neutral images. By observing individuals experiencing oral healthcare with positive outcomes, children may learn vicariously positive associations with dentistry.^13^ Alternatively the opportunity to view images of the dental surgery may serve to familiarize the child with the dental setting and serve as a form of preparatory information, such an approach has been shown to be effective in reducing anxiety in children undergoing dental surgery.

## LIMITATIONS

Positive and negative images used in the study were not validated. No attempt was made in the present study to identify the mechanism through which the reduction in anxiety had occurred, although this would be a fruitful area for future research. One putative mechanism for the effect noted would be a form of social learning through modeling.

## CONCLUSION

It is concluded that the effect of positive dental images differed from that of neutral images in reducing anxiety, when measured by the VPT. Hence, it was inferred that showing images when related to dental setting was an effective way of reducing anxiety in children.
